# The E3 ubiquitin ligase, RNF219, suppresses CNOT6L expression to exhibit antiproliferative activity

**DOI:** 10.1002/2211-5463.70081

**Published:** 2025-07-01

**Authors:** Shou Soeda, Melissa Montrose, Akinori Takahashi, Risa Ishida, Sandrine Burriel, Nao Ohmine, Tohru Natsume, Shungo Adachi, Minsoo Kim, Tadashi Yamamoto

**Affiliations:** ^1^ Cell Signal Unit Okinawa Institute of Science and Technology Japan; ^2^ Laboratory of Cell Systems Institute for Protein Research, Osaka University Japan; ^3^ Cellular and Molecular Biotechnology Research Institute (CMB), National Institute of Advanced Industrial Science and Technology (AIST) Tokyo Japan; ^4^ Laboratory of Integrative Molecular Medicine Graduate School of Medicine, Kyoto University Japan

**Keywords:** cancer, CCR4‐NOT complex, CNOT6L, deadenylation, RNF219, ubiquitin ligase

## Abstract

Despite the increasing evidence of the role of CCR4‐NOT complex in posttranscriptional gene regulation, relatively little is known about its mode of action. In a search for novel CCR4‐NOT interacting partners, we carried out mass spectrometry analysis of immunoprecipitates with antibodies against four different CCR4‐NOT subunits and identified RNF219, ring finger protein 219. A pull‐down assay revealed that the C‐terminal part of RNF219 directly binds to the CNOT1 DUF3819 domain and is associated with ubiquitin ligase activity. RNF219 knock‐down in HEK293T cells resulted in elevated expression of CNOT6L, accompanied by increased cell proliferation. The apparent antiproliferative activity of RNF219 was inversely correlated with the level of CNOT6L. Furthermore, RNF219 ubiquitinated CNOT6L *in vitro*. Our data suggest that RNF219 suppress CNOT6L expression through proteasome‐mediated protein degradation. Intriguingly, low expression of RNF219 was associated with poor prognosis of triple‐negative breast cancer patients. However, further studies would be required to confirm whether the impact of RNF219 activity on cancer progression is mediated by the CCR4‐NOT complex.

## Graphical abstract

We identified RNF219 as a CCR4‐NOT complex‐interacting E3 ubiquitin ligase that targets the CCR4‐NOT subunit CNOT6L for ubiquitination. RNF219 directly binds to the DUF3819 domain of CNOT1 through its putative α‐helix spanning amino acids 521–542. Our findings also suggest that antiproliferative activity of RNF219 is at least partially mediated by regulation of CNOT6L expression through ubiquitin‐proteasome‐mediated degradation.

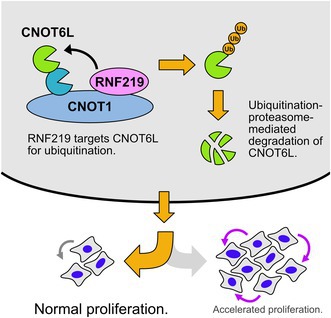

**Abbreviations**
IP‐MS: immune‐precipitation followed by mass spectrometry
SEM: standard errors of the means

In eukaryotes, gene expression is regulated both pre‐ and posttranscriptionally. Posttranscriptional control is exerted at the levels of splicing, translation, and mRNA decay. Although accumulating evidence supports the idea that the CCR4‐NOT complex is involved in these events [1, 2, 3], the molecular mechanisms involved are not fully understood.

The CCR4‐NOT complex is the major deadenylase in mammals for shortening of mRNA poly(A) tails, the initial step of mRNA degradation [4, 5, 6]. The mammalian CCR4‐NOT complex is composed of at least eight subunits, which include a central scaffold subunit, CNOT1, regulatory subunits, CNOT2, CNOT3, CNOT9, CNOT10, CNOT11, and a deadenylase core consisting of CNOT6 or CNOT6L and CNOT7 or CNOT8 [1]. CNOT2 and CNOT3 interact with the C‐terminal region of CNOT1 and regulate the stability and activity of the catalytic core [7, 8, 9]. CNOT10 and CNOT11 bind to the N‐terminal region of CNOT1 [10, 11]. CNOT9 docks on the central region of CNOT1, close to the catalytic core, and works as an adaptor for several RNA‐binding proteins, such as GW182 and tristetraprolin [12, 13, 14]. The catalytic core is composed of two types of subunits and possesses 3′–5′ exoribonuclease activity. CNOT7 and CNOT8 are DEDD‐type deadenylases, and they associate with the complex via CNOT1 [15, 16, 17, 18]. In contrast, CNOT6 and CNOT6L are EEP‐type deadenylases, and they associate with the complex via CNOT7 or CNOT8 [19, 20, 21, 22, 23, 24, 25, 26].

In addition to deadenylase, the yeast CCR4‐NOT complex contains another enzyme, E3 ubiquitin ligase Not4, which reportedly functions in translational quality control [27, 28]. Not4 interacts with ribosomes and ubiquitinates the Rps7A, which allows Not5 to interact with the E site of translating ribosomes, thereby bringing the CCR4‐NOT deadenylase into close proximity to target mRNAs [27, 29]. While this is an important step for translational quality control, it is unknown whether mammalian CNOT4 functions in a similar manner to the yeast Not4 homolog. Moreover, CNOT4 associates with CCR4‐NOT with very low affinity in humans and *Drosophila* [30, 31]. Another E3 ubiquitin ligase with RNA‐binding ability, MEX‐3C, is a mammalian CCR4‐NOT‐interacting protein [32]. MEX‐3C binds to the 3'UTR of HLA‐A2 mRNA, suppresses its translation, and regulates its levels by inducing its degradation [33]. Importantly, MEX‐3C ubiquitinates and activates the CNOT7 deadenylase subunit of the CCR4‐NOT complex [32]. Recent studies show that another RING domain‐containing protein, RNF219, a putative E3 ligase, interacts with the CCR4‐NOT complex to form mRNA‐associated granules and bodies [34, 35, 36]. As for physiological functions, several studies have reported that RNF219 is involved in regulation of stem cell differentiation [37], and in mild cognitive impairment, Alzheimer's disease, and treatment‐resistant depression [38, 39]. However, physiological functions of RNF219 in relation to the CCR4‐NOT complex are not well understood.

In this study, we discovered that RNF219 interacts with the DUF domain of the CNOT1 scaffold of the CCR4‐NOT complex. We further found that RNF219 exhibits antiproliferative activity by suppressing expression of CNOT6L through ubiquitination‐mediated protein degradation.

## Materials and methods

### Cell culture

HEK293T cells were cultured in DMEM (Thermo Fisher Scientific, Waltham, MA, USA) containing 10% fetal bovine serum, penicillin (50  U·mL^−1^), and streptomycin (50  U·mL^−1^) as described previously [40]. All cells were grown in a 5% CO_2_ atmosphere at 37 °C. MG132 was used at a concentration of 200 μm. In the growth assay using siRNA, cells were seeded in duplicate or triplicate in 6‐well or 12‐well plates. The number of cells was counted with a TC20™ Automated Cell Counter (BIO‐RAD, Hercules, CA, USA).

### Immunoblotting

Cells were solubilized in TNE buffer (50 mm Tris/HCl pH 7.5, 150 mm NaCl, 1 mm EDTA, 1% NP40, and 1 mm PMSF) for 30 min at 4 °C. Lysate dissolved in SDS sample buffer was subjected to SDS/polyacrylamide gel electrophoresis, followed by electro‐transfer onto Immobilon‐P membranes (Millipore, Barrington, MA, USA). Protein bands were blotted with primary antibodies CNOT1 (Biomatrix, Chiba, Japan, mouse monoclonal, custom‐made antibody), CNOT2 (Biomatrix, mouse monoclonal, custom‐made antibody), CNOT3 (Biomatrix, mouse monoclonal, custom‐made antibody), CNOT4 (Sigma, St. Louis, MO, USA, rabbit, HPA005737), CNOT6 (Biomatrix, mouse monoclonal, custom‐made antibody), CNOT6L (Biomatrix, mouse monoclonal, custom‐made antibody), CNOT7 (Biomatrix, mouse monoclonal, custom‐made antibody), CNOT8 (Biomatrix, mouse monoclonal, custom‐made antibody), CNOT9 (Biomatrix, mouse monoclonal, custom‐made antibody), CNOT10 (Abnova, Taipei City, Taiwan, mouse, H00025904‐B01), RNF219 (Invitrogen, Waltham, MA, USA, rabbit, PA5‐42338 or Bethyl, rabbit, A302‐540A), FLAG (Sigma, mouse, F1804), GST (MBL, Tokyo, Japan, rabbit, PM013, or CST, Danvers, MA, USA, rabbit, 2625S), MBP (NEB, Ipswich, MA, USA, mouse, E8032S, or CST, mouse, 2396S), T7 (MBL, rabbit, PM022), and Ubiquitin (Santa Cruz, Dallas, TX, USA, mouse, sc‐8017) and ECL anti‐rabbit or mouse IgG Horseradish Peroxidase (HRP)‐linked whole antibody (GE Healthcare, Chicago, IL, USA) as secondary antibodies. For detection, we used Immobilon Western HRP substrates (Millipore). To quantify the results, we used imagequant software in an Image Analyzer LAS 4000 mini (GE Healthcare Japan, Tokyo, Japan).

### Immunoprecipitation

HEK293T cells expressing FLAG‐tagged proteins were lysed with TNE lysis buffer (1% NP‐40, 50 mm Tris/HCl [pH 7.5], 150 mm NaCl, 1 mm EDTA, 1 mm phenylmethylsulfonylfluoride, and 10 mm NaF). Each lysate was incubated with anti‐FLAG antibody for 1 h at 4 °C with rotation, followed by incubation with Protein G Sepharose (GE Healthcare) for 2 h at 4 °C with rotation. Immunoprecipitated products were analyzed by immunoblotting.

### *In vitro* ubiquitination assay and *in vitro* pull‐down assay

MBP‐RNF219 proteins were expressed from the pML vector (GE) in *E. coli* (BL21) cells. MBP proteins were purified with Amylose Resin (NEB) and with Glutathione Sepharose 4B (GE), respectively. After MBP tag cleavage with PreScission Protease (GE), proteins were dialyzed against Ub assay buffer (10 mm Tris/HCl, 1 mm MgCl2, 4 mm DTT, 4 mm ATP, pH 7.5). For the ubiquitination assay, Ubiquitin‐activating enzyme E1 (Enzo), a series of E2 proteins (E2 Scan Kit; Cosmo Bio, Tokyo, Japan), purified recombinant RNF219, and Ubiquitin (Sigma) were incubated at 30 °C for 30 min in Ub assay buffer. For the CNOT6L ubiquitination assay, MBP‐RNF219 proteins and His‐T7‐CNOT6L were preincubated at 25 °C for 1 h before being mixed with Ubiquitin‐activating enzyme E1, E2 protein (UBE2D1), and ubiquitin. The reaction mixtures were incubated at 25 °C for 16 h in Ub assay buffer. Ubiquitination reactions were terminated by mixing with the same volume of SDS sample buffer (4% SDS, 0.25 m Tris, 0.4% 2‐mercaptoethenol, 20% glycerol, 0.02% BPB). For the *in vitro* pull‐down assay, purified recombinant MBP‐RNF219 proteins and purified recombinant His‐T7‐CNOT6L were incubated with Amylose Resin (NEB) or cOmplete His‐tag Purification Resin (Roche, Basel, Switzerland) at 4 °C for 4 h. Protein bead complexes were purified by centrifugation.

### Mass spectrometry followed by immunoprecipitation

Proteins were precipitated with anti‐CNOT2, ‐CNOT3, ‐CNOT6L, or CNOT9 antibodies from HEK293T cells as described above (immunoprecipitation), and precipitated proteins were eluted with 1%SDS. The eluted proteins were extracted by the methanol–chloroform protein precipitation method and precipitated proteins were re‐dissolved in guanidine hydrochloride, reduced with Tris(2‐carboxyethyl) phosphine hydrochloride, alkylated with iodoacetamide, and digested with lysyl endopeptidase and trypsin. The peptide mixture was applied to a Mightysil‐PR‐18 (Kanto Chemical, Tokyo, Japan) frit‐less column (45 × 0.150 mm ID) and separated using a 0–40% gradient of acetonitrile containing 0.1% formic acid for 80 min at a flow rate of 100 nL·min^−1^. The eluted peptides were sprayed directly into a mass spectrometer (Triple TOF 5600+; AB Sciex). MS and MS/MS spectra were obtained using the information‐dependent mode. Up to 25 precursor ions above an intensity threshold of 50 counts/s were selected for MS/MS analyses from each survey scan. All MS/MS spectra were searched against protein sequences of the RefSeq (NCBI) human protein database (RDB) using the protein pilot software package (AB Sciex, Tokyo, Japan), and decoy sequences were then selected with a false discovery rate < 1% [41].

### Statistical analyses

We used unpaired, two‐tailed Student's *t*‐tests. Values represent means ± standard errors of the means (SEM) and are represented as error bars. A *P*‐value < 0.05 was considered statistically significant.

## Results

### RNF219 binds directly to CNOT1 in the human CCR4‐NOT complex

**Fig. 1:**
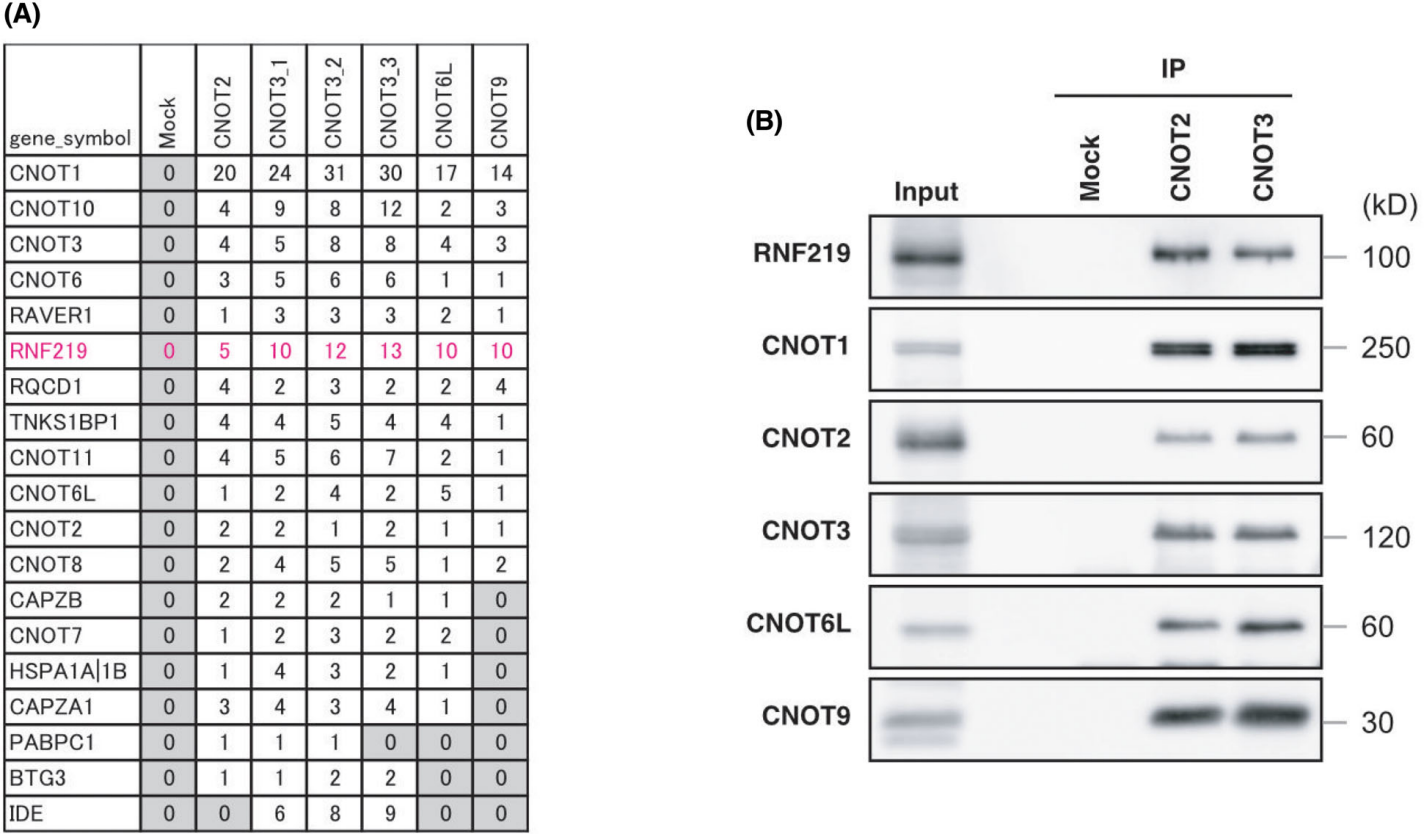
RNF219 is stable component of the CCR4‐NOT complex. (A) CCR4‐NOT complex and associated proteins were purified with anti‐CNOT2, CNOT3, CNOT6L, and CNOT9 antibodies from HEK293T cells. Immunoprecipitates were analyzed by mass spectrometry. Numbers of peptides detected are shown. (B) The CCR4‐NOT complex and associated proteins were purified with anti‐CNOT2 and CNOT3 antibodies from HEK293T cells. Immunoprecipitates were analyzed by western blotting.

While several studies have already reported RNF219 as an interacting partner of the CCR4‐NOT complex [35, 36, 37], we independently confirmed it with mass spectrometry (IP‐MS) of proteins precipitated with anti‐CNOT2, ‐CNOT3, ‐CNOT6L, or CNOT9 antibodies (Fig. 1A). RNF219 is decreased in the flow‐through fraction after CNOT3 IP, suggesting that a large proportion of RNF219 associates with the CCR4‐NOT complex. The interaction was confirmed by IP–western blotting (Fig. 1B).

**Fig. 2:**
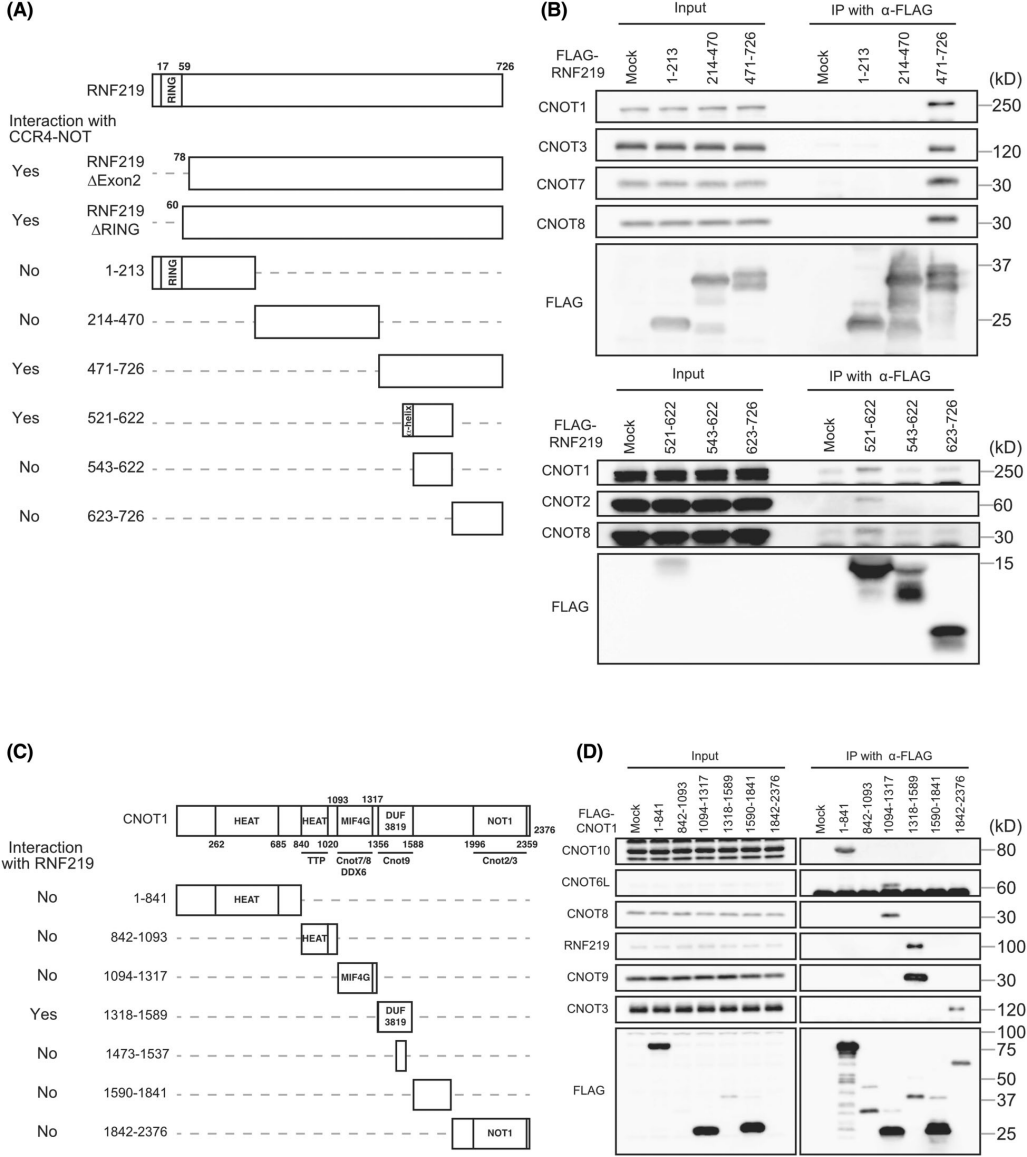
Identification of RNF219 and the CNOT1 peptide domain important for RNF219‐CCR4‐NOT complex interaction. (A) Schematic presentation of RNF219 fragments used in this experiment and result summary of interaction with the CCR4‐NOT complex. (B) Flag‐tagged RNF219 fragments were expressed in HEK293T cells and were purified with anti‐Flag antibody. Immunoprecipitates were analyzed by western blotting. (C) Schematic representation of CNOT1 fragments used in this experiment and a result summary of interaction with RNF219. (D) Flag‐tagged CNOT1 fragments were expressed in HEK293T cells and they were purified with anti‐Flag antibody. Immunoprecipitates were analyzed by western blotting.

To identify the binding mechanism of RNF219 with the CCR4‐NOT complex, we first scrutinized a domain of RNF219 that functions in its interaction with the CCR4‐NOT complex. A pull‐down assay with Flag‐tagged RNF219 fragments (Fig. 2A) showed that amino acids 521–542, in the C‐terminal half of RNF219, are largely responsible for its interaction with CNOT1, CNOT2, and CNOT8 (Fig. 2B).

**Fig. 3:**
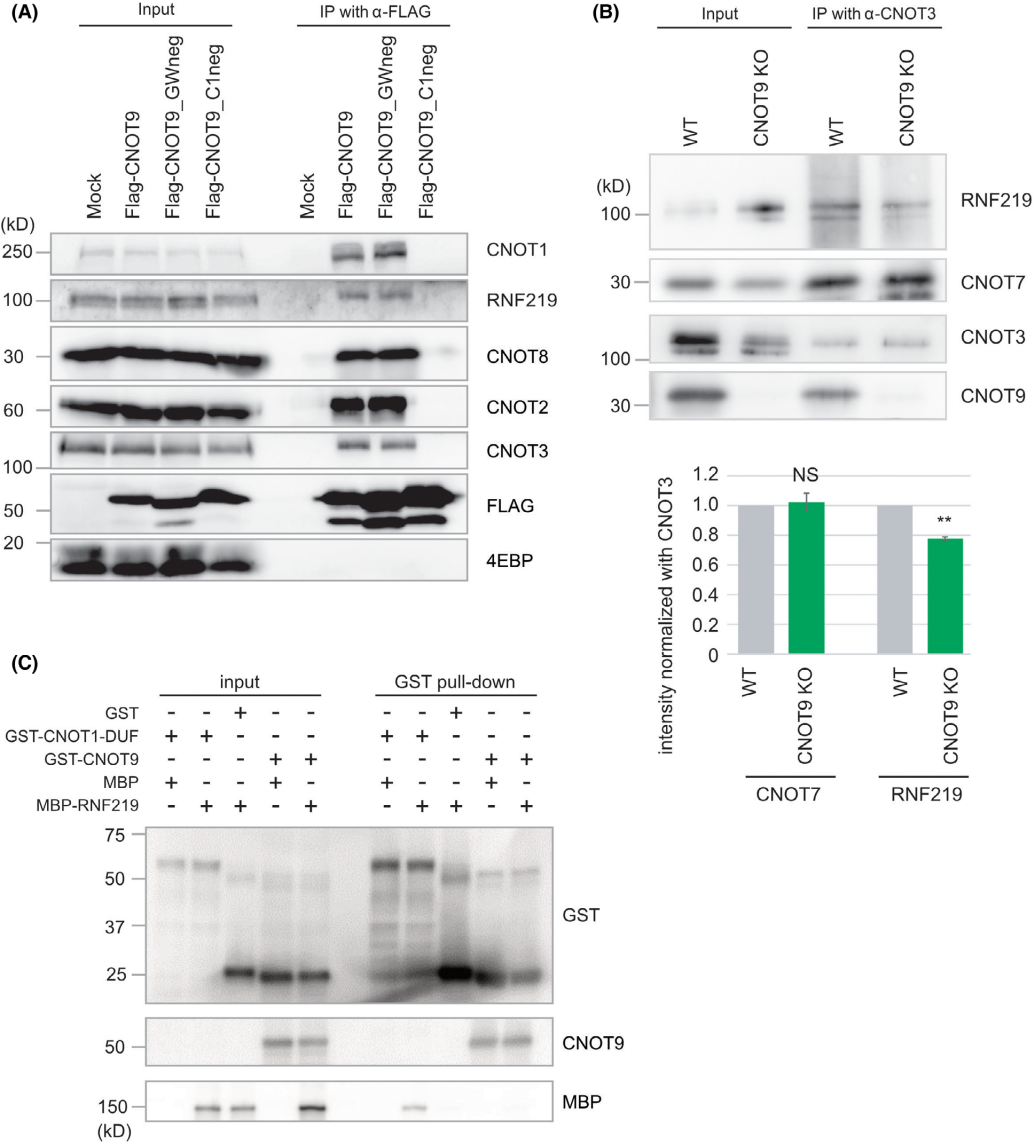
RNF219 binds directly to CNOT1 not via CNOT9. (A) Flag‐tagged CNOT9 WT, CNOT9_C1neg, or CNOT9_GWneg were expressed in HEK293T cells and they were purified with anti‐Flag antibody. Immunoprecipitates were analyzed by western blotting. (B) The CCR4‐NOT complex and associated proteins were purified with anti‐CNOT3 antibody from WT or CNOT9 KO HeLa cells. Immunoprecipitates were analyzed by western blotting. Signal intensity of CNOT7 or RNF219 was measured. Relative intensities are shown in the bar graph. Error bas: SEM. *n* = 3. Statistical significance by unpaired, two‐tailed Student's *t*‐tests is indicated (**P < 0.01, NS: *P* > 0.05). (C) Recombinant MBP‐RNF219, MBP, GST‐CNOT1‐DUF, GST‐CNOT9, and GST were purified from *E. coli*. After incubation, they were purified with GSH‐Sepharose beads. Eluates were analyzed by western blotting.

Next, to determine the interacting subunit within the CCR4‐NOT complex, we performed a pull‐down assay using cell lysates exogenously expressing a series of FLAG‐tagged CNOT1 fragments (Fig. 2C). Because CNOT1 is an interaction hub for other CCR4‐NOT complex subunits, identifying a CNOT1 domain important for interaction with RNF219 can help to identify the subunit that binds RNF219. RNF219 interacted with FLAG‐CNOT1 (1318–1589), a CNOT1 DUF3819 domain fragment (Fig. 2C,D). The DUF3819 domain also pulled down CNOT9, but not other subunits of the CCR4‐NOT complex (CNOT3, CNOT8, CNOT6L, and CNOT10) (Fig. 2D). FLAG‐CNOT1 (1473–1537), containing the middle of the CNOT DUF3819 domain, did not pull down either RNF219 or CNOT9 (Fig. 2D). To test whether RNF219 interaction with CNOT1 is mediated by CNOT9, we performed pull‐down assay with FLAG‐tagged CNOT9 mutants, which cannot bind to CNOT1 (CNOT9_C1neg) or GW182 (CNOT9_GWneg) [14]. Whereas CNOT9 WT and CNOT9_GWneg pulled down CNOT1 and RNF219 as well, CNOT9_C1neg failed to pull down either (Fig. 3A). This result suggests that association of RNF219 and CNOT9 may require CNOT1. In addition, the CCR4‐NOT complex immunoprecipitated from CNOT9 KO Hela cells with anti‐CNOT3 antibody contained RNF219, though the amount of RNF219 interacting with the CCR4‐NOT complex was decreased (Fig. 3B). Finally, to test the direct interaction between RNF219 and the CNOT1 DUF3819 domain, we performed an *in vitro* pull‐down assay with purified recombinant peptides. Purified peptides of the MBP‐RNF219 were pulled down by the GST‐CNOT1 DUF3819 domain, but not by GST‐CNOT9 (Fig. 3C). Thus, we concluded that RNF219 interacts directly with the CNOT1 DUF3819 domain, but not with CNOT9.

### E3 ubiquitin ligase activity of RNF219 affects its binding affinity to the CCR4‐NOT complex

**Fig. 4:**
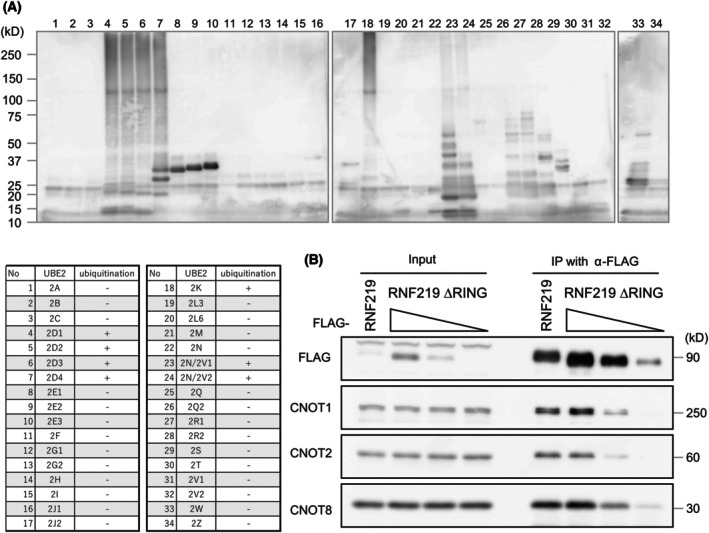
E3 ubiquitin ligase activity of RNF219 is important for binding of RNF219 to the CCR4‐NOT complex. (A) E2 scan assay (above) and result summary (below). Ube1, E2 enzymes, recombinant RNF219, and ubiquitin were incubated 1 h at 30 °C in the presence of ATP. After denaturing, they were analyzed by immunoblotting for ubiquitin. (B) Flag‐RNF219 WT or ∆RING was expressed in HEK293T cells. Flag‐RNF219 and associating CCR4‐NOT complex were purified with anti‐Flag antibody. Declining triangles indicate the expression level of RNF219 ∆RING. Immunoprecipitated proteins were analyzed by western blotting.

Recent studies have shown that RNF219 catalyzes ubiquitination [35]; however, E2 partners of RNF219 were not fully characterized. We conducted an *in vitro* ubiquitination assay using recombinant RNF219 protein (the amino‐terminal 161 amino acids that contains the ring finger domain) in the presence of 34 E2 subunits. RNF219 displayed ubiquitination activity with UBE2D1, 2, 3, 4, UBE2K, UBE2N/2 V1, and UBE2N/2 V2 (Fig. 4A).

Next, we addressed whether the ubiquitination activity of RNF219 affects the binding affinity to the CCR4‐NOT complex. Interestingly, although the RING domain of RNF219 was dispensable for its interaction with the CCR4‐NOT complex, the degree of interaction of RNF219 ΔRING mutant with the complex was reduced, compared to WT RNF219 (Fig. 4B). These data suggest that the RING domain of RNF219 is important for the binding affinity of RNF219 to the CCR4‐NOT complex.

### RNF219 suppresses cell growth by down‐regulation of the CNOT6L level

**Fig. 5:**
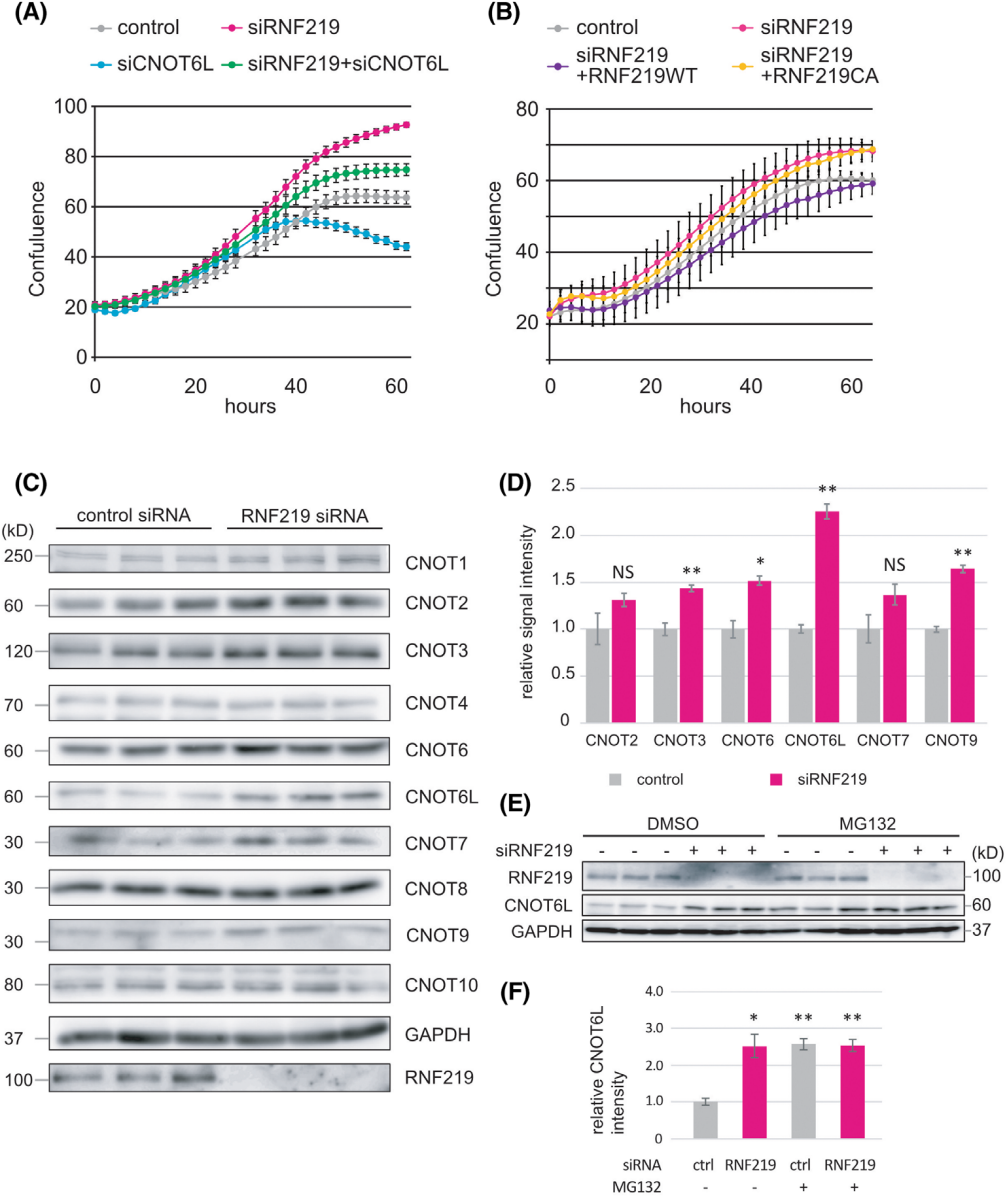
RNF219 exerts antiproliferative activity by suppressing CNOT6L expression. (A, B) Involvement of RNF219 and CNOT6L in cell proliferation. (A) HEK293T cells were transfected with siRNAs targeting RNF219 (siRNF219), CNOT6L (siCNOT6L), or both (siRNF219 + siCNOT6L), or non‐targeting siRNA (control). (B) HEK293T cells were transfected with siRNAs targeting RNF219 (siRNF219), or non‐targeting siRNA (control) followed by exogenous expression of siRNA‐resistant wild‐type RNF219 (RNF219WT), or RNF219 C21A mutant (RNF219CA). Cell proliferation was analyzed with an IncuCyte live‐cell analysis system. Error bars: SEM. (C, D) Expression of CCR4‐NOT complex subunits in RNF219 knock‐down cells. HEK293T cells were transfected with siRNA targeting RNF219 or non‐targeting siRNA. (C) Total cell lysates were analyzed by western blotting. Three biological replicates are shown. (D) Western blotting signal intensities were measured. Relative signal intensities are shown. Error bars: SEM. Statistical significance by unpaired, two‐tailed Student's *t*‐tests is indicated (*P < 0.05, **P < 0.01, NS: *P* > 0.05). (E) Effect of inhibition of proteasome‐mediated protein degradation by MG132. HEK293T cells transfected with siRNA targeting RNF219 or non‐targeting siRNA were treated with MG132 or DMSO for 8 h. Total cell lysates were analyzed by western blotting. Three biological replicates are shown. (F) CNOT6L signal intensities were measured. Relative signal intensities are shown. Error bars: SEM. Statistical significance by unpaired, two‐tailed Student's *t*‐tests is indicated (*P < 0.05, **P < 0.01).

**Fig. 6:**
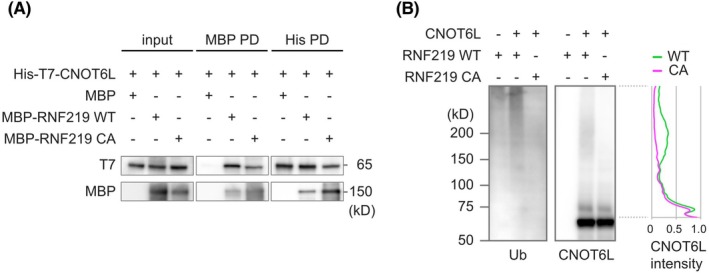
RNF219 ubiquitinates CNOT6L *in vitro*. (A) *In vitro* pull‐down assay with recombinant RNF219 and CNOT6L. Association between MBP‐RNF219 (WT or C21A mutant) and His‐T7‐CNOT6L were analyzed by MBP tag pull‐down (MBP PD) or by His‐tag pull‐down (His PD). Co‐purified proteins were analyzed by western blotting. (B) *In vitro* ubiquitination assay with recombinant RNF219 and CNOT6L. After ubiquitination reaction, proteins were analyzed by western blotting. CNOT6L intensities were quantified and shown in the right graph.

We investigated the physiological relevance of RNF219 suppression by knock‐down experiments. RNF219 siRNA‐treated cells showed increased proliferation, compared with control cells (Fig. 5A). Exogenous expression of siRNA‐resistant wild‐type RNF219 suppressed increased proliferation in siRNF219‐treated cells, whereas the C21A RING domain mutant, which lacks E3 ubiquitin ligase activity, did not (Fig. 5B), suggesting that RNF219 exerts antiproliferative activity by suppressing gene expression via proteasome‐mediated degradation. To address how RNF219 suppresses cell proliferation, we sought the proteins that are upregulated in RNF219 knock‐down cells. We started by searching CCR4‐NOT components, expression levels of which were altered by RNF219 knock‐down. Western blotting experiments showed that levels of CNOT2, CNOT3, CNOT6, CNOT6L, and CNOT9 proteins were significantly increased (Fig. 5C). The increase of CNOT6L protein expression was especially impressive (Fig. 5D). The pro‐proliferative nature of CNOT6L was suggested, as its suppression resulted in growth retardation of both mouse cells [42] and human cells [43]. Here we also showed that CNOT6L knock‐down suppressed proliferation of HEK293T cells (Fig. 5A). Importantly, cell growth suppressed by CNOT6L knock‐down was reversed by concomitant knock‐down of RNF219 (Fig. 5A). These results suggest that the antiproliferative activity of RNF219 is partially mediated by regulation of CNOT6L expression. To address the mechanism by which RNF219 regulates CNOT6L expression, we investigated the involvement of ubiquitin‐proteasome‐mediated protein degradation. CNOT6L expression was significantly increased after the proteasome inhibitor, MG132 treatment (Fig. 5E, F), suggesting that CNOT6L is labile to a ubiquitin‐proteasome mechanism. Note that the CNOT6L level in RNF219 knock‐down cells was affected only a little by the presence of the proteasome inhibitor MG132 (Fig. 5E, F). Furthermore, we tested whether RNF219 directly ubiquitinates CNOT6L. *In vitro* pull‐down assay showed direct interaction between CNOT6L and both RNF219 WT and the C21A mutant, which lacks E3 ubiquitin ligase activity (Fig. 6A). We next performed an *in vitro* ubiquitination assay. CNOT6L showed low mobility signals, an indicator of ubiquitination when it was reacted with RNF219 WT, whereas the signal was not observed with the RNF219 C21A (Fig. 6B). The results indicate that CNOT6L is one of the ubiquitination targets for RNF219. Taken together, the above observations indicate that RNF219 ubiquitinates CNOT6L and thereby regulates its protein expression through ubiquitin‐proteasome‐mediated protein degradation.

Since our data suggested that RNF219 is antiproliferative, we examined RNF219 expression in cancers. By scrutinizing the cancer genome atlas database, we found that RNF219 expression was significantly decreased in malignant tissues of patients with cancers of breast, brain, prostate, thyroid, thymus, and uterus (Fig. S1A). Next, we examined whether RNF219 expression level is related to breast cancer prognosis. Kaplan–Meier plots with classifications based on RNF219 mRNA expression level revealed that survival of patients with triple‐negative breast cancer was poor (Fig. S1B) [44]. Therefore, we assumed that RNF219 expression level is correlated with malignancy of breast cancer.

## Discussion

We identified RNF219 as a CCR4‐NOT complex‐interacting E3 ubiquitin ligase. RNF219 binds the CNOT1 DUF3819 domain through its putative α‐helix at amino acids 521–542. Interestingly, an α‐helix of CNOT9 also binds the CNOT1 DUF3819 domain [14]. Although another group [36] showed that RNF219 and CNOT9 might associate cooperatively with the CCR4‐NOT complex, we showed here that the RNF219 association with CNOT1 does not require CNOT9. On the other hands, our results showed that RNF219 expression was increased in CNOT9 KO cells and that the association of RNF219 with CCR4‐NOT complex was decreased in CNOT9 KO cells (Fig. 3B). These results suggest that CNOT9 may regulate the interaction between RNF219 and CNOT1 and thereby regulate the stability of RNF219.

RNF219 can work together with E2 enzymes, UBE2Ds, UBE2K, and UBE2Ns. These E2 enzymes and RNF219 localize to the cytoplasm [45], and in this study, we showed that RNF219 suppresses CNOT6L protein expression through proteasome‐mediated protein degradation. Despite the high similarity, CNOT6 was not highly upregulated compared to CNOT6L (Fig. 5C,D). The competition between CNOT7 and CNOT8 for integration into the CCR4‐NOT complex leads to the difference in their stability [46]. Similarly, if CNOT6 and CNOT6L have different affinities for the complex, this could lead to different levels of effects on their stability in RNF219‐depleted cells. Suppression of CNOT6L by RNA interference results in growth retardation of NIH 3 T3 cells, accompanied by elevation of both p27Kip1 mRNA and Kip1 protein [42]. Thus, the current observation that RNF219 disruption results in stimulation of cell proliferation might be due at least in part to prevention of ubiquitination‐mediated CNOT6L degradation. As RNF219 and partner E2 enzymes also localize in the nucleus [45], nuclear RNF219‐mediated polyubiquitination of its putative nuclear target may also contribute to regulation of cell proliferation.

Our findings suggest that ubiquitination of the CCR4‐NOT subunit CNOT6L is relevant to cell growth control. Intriguingly, there is a report that another CCR4‐NOT subunit CNOT7 is also ubiquitinated by another E3 ubiquitin ligase, MEX‐3C. In this case, proteolytic degradation of CNOT7 is not induced. Instead, CNOT7 becomes activated by ubiquitination, which leads to deadenylation and degradation of major histocompatibility complex‐1 mRNA [32].

Not4, a yeast homolog of CNOT4, is a ubiquitin ligase associated with the CCR4‐NOT complex. In contrast, mammalian CNOT4 is not stably associated with the complex. Not4 protein functions in translation quality control through ubiquitination of S7 [47]. Although a functional mammalian Not4 counterpart in the context of quality control is uncertain, mouse CNOT4 also appears to ubiquitinate S7 [48]. Although protein sequence identity of RNF219 to CNOT4 is less than 15%, it would be interesting to test whether mammalian RNF219 has functions similar to those of yeast Not4.

We found that RNF219 is antiproliferative and is expressed at relatively low levels in malignant triple‐negative breast cancer; however, it is unclear whether the low expression level of RNF219 is a cause or a result of malignancy. Nonetheless, it might be possible to apply this finding to predict cancer prognoses. Control of mRNA stability by the CCR4‐CNOT complex is associated with oncogenesis [49, 50]. Although we demonstrate that antiproliferative activity of RNF219 is at least partially mediated by regulation of CNOT6L expression, further investigation would be required to conclude that the impact of RNF219 activity on cancer progression is mediated by the CCR4‐NOT complex.

Consistent with our study, a recent independent investigation also shows that RNF219 is a CCR4‐NOT interacting E3 ubiquitin ligase [34, 35, 36, 37]. Our own characterization of RNF219 as an E3 ligase, discovery of its cooperation with UBE2D1–4, UBE2K, and UBE2N/2 V1‐2 in a complex, and identification of CNOT6L as one of its important ubiquitination targets are unique and should help better understand the biological significance of its association with the CCR4‐NOT complex.

## Conflict of interest

The authors declare no conflict of interest.

## Peer review

The peer review history for this article is available at https://www.webofscience.com/api/gateway/wos/peer‐review/10.1002/2211‐5463.70081.

## Author contributions

SS, AT, and TY designed the research. TN and SA performed mass spectrometry analysis. RI and MK performed *in vitro* ubiquitination assay. SS, MM, AT, SB, and NO performed all other experiments and data analysis. SS and TY wrote the manuscript. All authors have read and agreed to the published version of the manuscript.

## Supporting information


**Fig. S1.** RNF219 expression level is related to cancer prognosis. (A) RNF219 expression level in tumor tissue. RNF219 mRNA expression level was compared in 22 normal and tumor tissues. mRNA expression data is from The Cancer Genome Atlas database. Tissues in which RNF219 levels are significantly low in tumors are shown. (B) Kaplan–Meier plot of the patient survival rate of triple‐negative breast cancer. The patient survival rate was compared in the high (*n* = 113) and low (*n* = 279) RNF219 expression groups. The analysis was done with a KM plotter [50].
