# Supplementary material for: The E3 ubiquitin ligase, RNF219, suppresses CNOT6L expression to exhibit antiproliferative activity
Source: FEBS Open Bio. 2025 Jul 1;15(11):1874–85. doi: 10.1002/2211-5463.70081 (PMC12582992; doi:10.1002/2211-5463.70081)
Supplement: Supplementary file 1 — Fig. S1. RNF219 expression level is related to cancer prognosis. (A) RNF219 expression level in tumor tissue. RNF219 mRNA expression level was compared in 22 normal and tumor tissues. mRNA expression data is from The Cancer Genome Atlas database. Tissues in which RNF219 levels are significantly low in tumors are shown. (B) Kaplan–Meier plot of the patient survival rate of triple‐negative breast cancer. The patient survival rate was compared in the high (n = 113) and low (n = 279) RNF219 expression groups. The analysis was done with a KM plotter [50]. [file FEB4-15-1874-s001.pdf]

Fig. S1

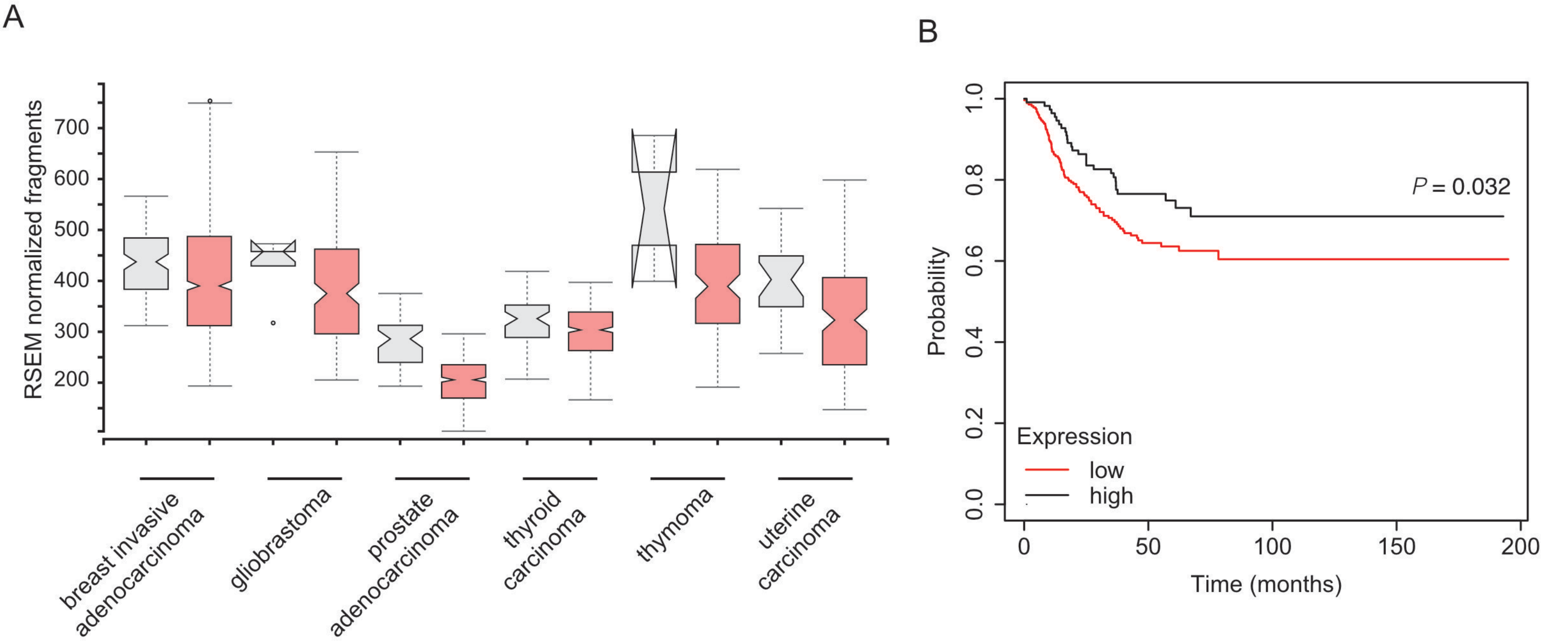

Supplementary Figure 1. RNF219 expression level is related to cancer prognosis. (A) RNF219 expression level in tumor tissue. RNF219 mRNA expression level was compared in 22 normal and tumor tissues. mRNA expression data is from The Cancer Genome Atlas database. Tissues in which RNF219 levels are significantly low in tumors are shown. (B) Kaplan-Mayer plot of the patient survival rate of triple-negative breast cancer. The patient survival rate was compared in the high (n = 113) and low (n = 279) RNF219 expression groups. The analysis was done with a KM plotter [50].
